# Editorial: Advances and challenges in studying brain disorders: from development to aging

**DOI:** 10.3389/fnins.2024.1341410

**Published:** 2024-01-29

**Authors:** Esther Klingler, Subashika Govindan, Fiona Francis

**Affiliations:** ^1^Department of Neuroscience, VIB-KU Leuven Center for Brain and Disease Research, Leuven, Belgium; ^2^Leuven Brain Institute, KU Leuven, Leuven, Belgium; ^3^Wellcome Trust/DBT - India Alliance, Hyderabad, India; ^4^Indian Institute of Technology - Madras, Chennai, Tamil Nadu, India; ^5^Institut du Fer à Moulin, Paris, France; ^6^Institut National de Santé et de Recherche Médicale (INSERM) UMR-S 1270, Paris, France; ^7^Sorbonne Universités, Paris, France

**Keywords:** brain development, brain aging, neurodegenerative diseases, neurodevelopmental diseases, intrinsic - extrinsic, cell-type susceptibilities, genetic mutation, *in vitro in vivo*

Development and function of the human brain depends on multiple molecular programs, the fine balance of heterogeneous cell types and coordination of distinct anatomical structures. The human brain is vulnerable to a range of diseases that manifest at different life stages, affecting various cell types and structures. Despite the protection provided by the blood-brain barrier in adulthood, neurons, the fundamental unit of the brain, which receive, integrate and transmit signals, are highly susceptible to developmental and cumulative defects or injuries due to very limited renewal. Although brain disorders are traditionally categorized as “neurodevelopmental,” “neuropsychiatric,” and “neurodegenerative,” there is some evidence that these conditions share common molecular and cellular pathways with diverse outcomes based on region- and cell-type-specific susceptibilities. In this Research Topic, we have compiled hypotheses and research articles, along with reviews, to shed light on current and complementary approaches of studying brain diseases from development to aging.

Brain development relies on the production of neurons from neural progenitor cells, which, when affected, lead to various neurodevelopmental disorders. For instance, abnormal regulation of neural progenitor polarity can lead to not only abnormal division, neuron production and microcephaly, but also to epilepsy and/or autism spectrum disorders (ASD). These defects can stem from mutations in genes coding for trafficking-related structural proteins and enzymes within the endoplasmic reticulum and the Golgi apparatus of progenitors, as discussed in Polenghi and Taverna.

Once neurons are generated, their migration, differentiation, and establishment of connections are crucial for correctly forming functional circuits. Identifying the developmental origins of neurons in a specific brain region is essential for fully understanding connectivity and function, in both normal and diseased conditions. For example, neurons of distinct parts of the *substantia nigra pars reticulata* (SNpr) have different connectivities and are differentially involved in seizures. Those originating from diencephalon/midbrain respond to short-term flexible reward, while neurons originating from the hindbrain and colonizing a more posterior part of the SNpr, play a role in long-term high-value reward encoding (Partanen and Achim). Establishment of proper brain connectivity also relies on precise controls over gene expression levels. For example, different levels of the Caspr2 protein encoded by the *CNTNAP2* gene (whose mutations can lead to several brain disorders, from neurodevelopmental to peripheral neuropathies) lead to different abnormalities in connectivity tracts. In mice, *Cntnap2* heterozygosity can even lead to a stronger phenotype than null homozygosity in the case of callosal axon diameters or cortical neuron intrinsic excitability (Cifuentes-Diaz et al.). With respect to disease causing mutations, a diversity of phenotypes can also occur even with a single pathogenic variant: patients with the *NHLRC2* pathogenic variant c.442G > T (either homozygous or heterozygous) have various defects in multiple organs (including the brain) affecting development and function, and leading to Fibrosis, Neurodegeneration and Cerebral Angiomatosis (FINCA) (Tallgren et al.). The FINCA disease is a good example of how perturbation of single molecule can manifest both with neurodevelopmental and neurodegenerative phenotypes.

It is not only cell origins which are important in defining cell development and later function, but also the changes which can arise after birth. Somatic mutations occur throughout cell life and particularly affect brain functions. These have been implicated in several brain disorders, from developmental, such as focal cortical dysplasia, autism spectrum disorders and schizophrenia, to neurodegenerative conditions, such as Alzheimer's disease. In her review, Bizzotto explores cutting edge discoveries from the last 5 years that utilize single-cell and next generation sequencing, and sheds light on how accumulation of somatic mutations in aging brain cells can inform on cell-type-specific disease predisposition.

The correct production, migration, growth of axons and synapse development of neurons are also all sensitive to extrinsic factors, e.g., via immune-responsive cell types, such as microglia. Microglia, originating outside the central nervous system, invade the brain during early embryogenesis, before any other glial cell is present, and influence both brain development and later function. Their involvement has been associated with various brain disorders, including ASD, schizophrenia, and neurodegenerative diseases such as Alzheimer's disease, as discussed by Bridlance and Thion. To further explore the effect of environmental factors such as viral infections on brain function, Mahajan et al. show that the loss of function of Angiotensin Converting Enzyme-2 (ACE2) receptor, one of the key molecular factors mediating SARS-CoV-2 entry into the central nervous system, leads to distinct morphological changes, abnormal odor processing and associated cognitive disability.

In summary, a diversity of mechanisms can contribute to a single disease, and distinct deficits in one specific cell type can contribute to a range of brain diseases, which highlights the need to strengthen the bridge between neurodevelopmental and neurodegenerative studies. This can be achieved through cross-comparisons of animal models and different datasets to uncover common and specific mechanisms/pathways, aiding in drug discovery. For example, Grünblatt et al. emphasize the links between Alzheimer's disease and attention deficit hyperactivity disorder (ADHD), proposing a common dysregulation of molecular pathways involving the Wnt/mTOR pathway at different life stages. Additionally, leveraging transcriptomics datasets allows for the comparison of disease-related pathways, as demonstrated by Ye et al., who provide new insights on the immune microenvironment, but also the transcription factors and miRNA regulation networks at play in neuropathic pain and aging animal models. Finally, using *in vitro* technologies, such as induced pluripotent stem (iPS) cells derived from patients, also provides new avenues for modeling brain diseases, as discussed by Loussert-Fonta et al. in their approach to model traumatic brain injury in a dish.

Altogether, our Research Topic elucidates the multifaceted etiology of numerous brain disorders, converging on a range of factors, from point mutations at the genome level, to gene expression changes at the molecular level, to perturbation at the cellular and anatomical level, to environmental factors such as infectious diseases and immune responses. This Research Topic further reveals how we can leverage on latest cutting-edge technologies such as next generation sequencing, single-cell sequencing, CRISPR-Cas9 based gene editing, iPS technologies and tissue engineering to put ourselves in the forefront of studying brain disorders.

## Author contributions

EK: Writing – original draft. SG: Writing – review & editing. FF: Writing – review & editing.

